# Family history and risk of ductal carcinoma *in situ* and triple negative breast cancer in a Han Chinese population: a case–control study

**DOI:** 10.1186/1477-7819-11-248

**Published:** 2013-10-01

**Authors:** Wenbin Zhou, Hong Pan, Mengdi Liang, Kai Xia, Xiuqing Liang, Jinqiu Xue, Lin Cheng, Jialei Xue, Si Chen, Xiaoan Liu, Qiang Ding, Lijun Ling, Shui Wang

**Affiliations:** 1 Department of Breast Surgery, The First Affiliated Hospital with Nanjing Medical University, 300 Guangzhou Road, Nanjing 210029, China; 2 Department of Breast Surgery, The Affiliated Jiangyin Hospital of Southeast University Medical College, 163 Shoushan Road, Jiangyin 214400, China

**Keywords:** Case–control, Ductal carcinoma *in situ*, Family history, Triple negative breast cancer

## Abstract

**Background:**

The association between family history and risk of triple negative breast cancer and ductal carcinoma *in situ* (DCIS) has not been well investigated, especially in Asian populations. We investigated the association between family history and risk of DCIS or triple negative breast cancer in a Han Chinese population.

**Methods:**

A case–control study, comprising 926 breast cancer patients and 1,187 benign breast disease controls, was conducted in our hospital. Multivariate logistic regression was used to assess the relationships between family history and risk of DCIS or triple negative breast cancer.

**Results:**

Subjects with a family history of breast cancer had higher breast cancer risk than those without a family history (odds ratio (OR) = 2.11, 95% confidence interval (CI) = 1.26 to 3.52). Family history was not significantly associated with an increased risk of DCIS (OR = 1.27, 95% CI = 0.36 to 4.46), while family history was significantly associated with an increased risk of invasive breast cancer (OR = 2.22, 95% CI = 1.32 to 3.75), irrespective of triple negative breast cancer (OR = 3.35, 95% CI = 1.43 to 7.88) or non-triple negative breast cancer (OR = 2.14, 95% CI = 1.21 to 3.80).

**Conclusion:**

Our results indicate that having a family history of breast cancer is associated with an increased risk of triple negative breast cancer with a magnitude of association similar to that for non-triple negative breast cancer. Furthermore, family history is not significantly associated with an increased risk of DCIS. Future cohort studies with larger sample sizes are still needed to explore these relationships.

## Background

Breast cancer is a worldwide malignant disease, and it is the leading cause of cancer-related death in women [1,2]. There is plentiful evidence to indicate that breast cancer is an epidemiologically, biologically, and clinically heterogeneous disease [3]. A special subgroup of patients is associated with aggressive clinical behavior and poor outcome [4-6]. This subgroup of breast cancer is characterized by absent expression of estrogen receptor (ER), progesterone receptor (PR) and HER2 receptor and is defined as triple negative breast cancer.

To the authors’ knowledge, few risk factors for this breast cancer subtype have been identified. Family history is a key risk factor of breast cancer [7-12]. However, the association between family history and risk of triple negative breast cancer has not been well investigated. Some studies suggest that there is no difference in the prevalence of family history between women with triple negative breast cancer (or basal-like breast cancer) and other types of breast cancer [3,13]. By contrast, another previous study [14] suggests that a family history was associated with an increased risk of basal-like breast cancer. Furthermore, Jiang and colleagues observed an increased proportion of ER and PR negative breast cancer among younger Spanish women with a family history of breast cancer [15]. Therefore, the relationship between family history and risk of triple negative breast cancer is still not clear.

The incidence and mortality of breast cancer are significantly affected by racial or ethnic difference [15-19]. For example, previous studies suggested that Hispanic women had a lower rate of breast cancer than non-Hispanic white women, while Hispanic breast cancer patients were more likely to die of breast cancer than non-Hispanic white patients [15,20]. Furthermore, about 20% of Hispanic invasive breast cancer patients had a family history [15], while the proportion of breast cancer patients with a family history was only about 6.6% in a cross-sectional survey in China [1]. Up to now, there are limited amounts of data on the association between family history and triple negative breast cancer risk in Asian populations. Furthermore, there is little data on the relationship between family history and risk of ductal carcinoma *in situ* (DCIS) [21-25]. However, there is no data on the association between family history and risk of DCIS in Asian populations.

The aim of this study was to investigate the association between family history and risk of DCIS or triple negative breast cancer in a Han Chinese population. To our knowledge, this case–control study represents one of the first studies to determine these relationships in a large population of Chinese women.

## Methods

### Ethics

The study was approved by the ethics committee of the First Affiliated Hospital with Nanjing Medical University, and was performed in compliance with the Helsinki Declaration. All patients provided written informed consent for their clinical data to be reviewed by us.

### Study population

Women with histologically confirmed breast cancers, on whom surgeries were performed by one group of surgeons in the First Affiliated Hospital with Nanjing Medical University from May 2005 to July 2012 were recruited as the case group. All subjects in this study were diagnosed with incident breast cancer, and were recruited without restriction of age or histological type. The patients were ethnic Han Chinese coming from different families. Patients with previous cancer history, metastasized cancer from other organs, and previous chemotherapy or radiotherapy were excluded. A control group of subjects with histologically confirmed benign breast disease, on whom surgeries were performed by the same group of surgeons in the same period, was also recruited. Both groups of patients were recruited without restriction of age, and were all ethnic Han Chinese, from different families without previous cancer history.

### Data collection

The database of breast disease (breast cancer and benign breast disease) in our hospital was reviewed. Family history was defined as a history of breast cancer in any first-degree or second-degree relative [15]. Risk factors and clinical information were collected from medical records by trained physicians. The following selected risk factors were extracted if available: age at diagnosis, age at menarche, previous childbearing history, and family history of breast cancer. The following clinicopathologic variables were selected: pathology, tumor size, number of positive lymph nodes, ER status, PR status, and HER2 status.

### Pathology

For histopathological examinations, the specimens were paraffin-embedded. Then, 4 μm histological sections were cut and stained with H & E. Immunohistochemical analyses on paraffin-embedded material were used to determine the ER and PR status, as described previously [26]. HER2 status was determined according to the American Society of Clinical Oncology guidelines [27]. DCIS with microinvasion ≤1 mm was used as the DCIS category. All patients with invasive breast cancer in this study were categorized into two subgroups: triple negative and non-triple negative. Either or both of ER and PR positive were considered hormone receptor positive, while both ER and PR negative were considered hormone receptor negative. Triple negative breast cancer was defined as ER, PR, and HER2 all negative. The pathology for both case and control groups was reviewed independently by an experienced pathologist.

### Statistical analysis

Percentiles were used to analyze for numerical variables, and mean ± standard deviation (SD) was also used to report the numerical data. Student’s *t* test was applied to identify the difference of mean age between the case and control groups. The chi-square test was applied to examine the differences for unordered categorical variables between the case and control groups, while the nonparametric rank test was applied to examine the differences for ordinal categorical variables between the two groups. Multivariate logistic regression analyses were performed to identify risk factors of breast cancer and subtype-specific breast cancer. The candidate explanatory variables in the multivariate logistic regression analysis were: age at diagnosis, age at menarche, previous childbearing history, and family history of breast cancer. Importantly, those with a family history of breast cancer are often diagnosed much younger than those without a family history. Age at diagnosis can therefore be an intermediate between family history and the occurrence of breast cancer. Adjusting for a disease intermediate can result in bias away from the null. So we also ran these analyses without age at diagnosis to see how much this affected the odds ratio (OR). Age at diagnosis in this study was categorized into seven subgroups: ≤30, 31 to 40, 41 to 50, 51 to 60, 61 to 70, 71 to 80, and ≥81. Age at menarche was categorized into three subgroups: ≤13, 14 to 16, and ≥17. The number of children carried in this study was categorized into four subgroups: 0, 1, 2, and ≥3. Odds ratios and 95% confidence interval (CI) were used to assess breast cancer risk of candidate explanatory variables. All statistical analyses were performed using Stata version 11.0 (StataCorp, College Station, Texas). All tests were two-sided and a level of significance of 0.05 was applied.

## Results

A total of 926 women with breast cancer and 1,187 women with benign breast disease were studied. The selected characteristics for breast cancer patients and control subjects are presented in Table 1. The mean age of the control group (44.8 ± 12.5) was younger than that of the case group (52.2 ± 12.2) (*P* < 0.001). Age at menarche was not well balanced between these two groups (*P* < 0.001). Furthermore, more patients in the case group had two or more children (30.6%) than in the control group (19.4%) (*P* < 0.001); this was in accordance with the age distribution between the two groups. In this study population, 5.1% of breast cancer patients had a family history of breast cancer, which was higher than that in control group (3.2%) (*P* = 0.012).

**Table 1:** Distribution of selected variables between breast cancer patients and control subjects

**Variable**	**Cases (*n *= 926)**		**Controls (*n *= 1,187)**		***P***^**a**^
	***N***	**%**	***N***	**%**	
Age at diagnosis (mean ± SD), years	52.2 ± 12.2		44.8 ± 12.5		<0.001
≤50	453	48.9	819	69.0	<0.001
>50	473	51.1	368	31.0	
Age at menarche, years					
≤13	144	15.6	229	19.3	<0.001
14 to 16	406	43.8	643	54.2	
≥17	138	14.9	114	9.6	
Not available	238	25.7	201	16.9	
Childbearing history					
0 children	26	2.8	132	11.1	<0.001
1 child	456	49.2	707	59.6	
2 children	191	20.6	162	13.6	
≥3 children	93	10.0	69	5.8	
Not available	160	17.3	117	9.9	
Family history					
Yes	47	5.1	38	3.2	0.012
No	814	87.9	1143	96.3	
Not available	65	7.0	6	0.5	

^a^Student’s *t* test for age distribution between case and control groups; chi-square test for family history distribution between case and control groups; nonparametric rank test for other variables distribution between case and control groups.

### Multivariate analysis of breast cancer risk

In multivariate logistic analyses (Table 2), age at menarche (all *P* > 0.1) and previous childbearing history (all *P* > 0.1) were not significantly associated with an increased or decreased breast cancer risk when other variables were adjusted. Compared with subjects younger than 30 years, increased age was significantly associated with increased breast cancer risk in all age subgroups (31 to 40 y: OR = 6.14, 95% CI = 2.58 to 14.64; 41 to 50 y: OR = 10.31, 95% CI = 4.34 to 24.51; 51 to 60 y: OR = 14.46, 95% CI = 6.02 to 34.69; 61 to 70 y: OR = 16.08, 95% CI = 6.41 to 40.33; 71 to 80 y: OR = 31.12, 95% CI = 11.69 to 82.89; ≥81 y: OR = 38.86, 95% CI = 6.30 to 239.57). When other variables were adjusted, subjects with a breast cancer family history had a higher breast cancer risk than subjects without a family history (OR = 2.11, 95% CI = 1.26 to 3.52, *P* = 0.004). Similar results were obtained (OR = 2.15, 95% CI = 1.31 to 3.53, *P* = 0.003) when multivariate analysis was performed without age at diagnosis.

**Table 2:** Multivariate logistic analysis of breast cancer-related factors compared with benign breast disease control

**Variable**	**OR**	**95% CI**	***P***
Age at diagnosis, years			
≤30	Reference		
31 to 40	6.14	2.58 to 14.64	<0.001
41 to 50	10.31	4.34 to 24.51	<0.001
51 to 60	14.46	6.02 to 34.69	<0.001
61 to 70	16.08	6.41 to 40.33	<0.001
71 to 80	31.12	11.69 to 82.89	<0.001
≥81	38.86	6.30 to 239.57	<0.001
Age at menarche, years			
≤13	Reference		
14 to 16	0.83	0.63 to 1.09	0.172
≥17	1.21	0.84 to 1.74	0.309
Childbearing			
0	Reference		
1	1.24	0.71 to 2.16	0.448
2	1.52	0.84 to 2.76	0.170
≥3	1.47	0.75 to 2.89	0.226
Family history			
No	Reference		
Yes	2.11	1.26 to 3.52	0.004

CI, confidence interval; OR, odds ratio.

### Comparison between triple negative and non-triple negative breast cancer

Of 926 breast cancer patients in this study, 123 patients were diagnosed with DCIS, and 803 were diagnosed with invasive cancer. Of these 803 invasive cancer patients, ER, PR and HER2 status were all available in 706 patients, including 129 triple negative breast cancer patients, and 577 non-triple negative breast cancer patients. The distribution of selected variables between triple negative and non-triple negative breast cancer patients is shown in Table 3. Age at diagnosis (*P* = 0.354), age at menarche (*P* = 0.494), and previous childbearing history (*P* = 0.934) were not significantly different between triple negative and non-triple negative patients. Furthermore, tumor size between the two groups was not significantly different (*P* = 0.792). Like our previous study [26], triple negative breast cancer (34.88%) had less lymph node involvement than non-triple negative breast cancer (50.26%) (*P* = 0.001). Importantly, the proportions of patients with a family history between the two groups were not significantly different (*P* = 0.599).

**Table 3:** Distribution of selected variables between triple negative and non-triple negative breast cancer patients

**Variable**	**Triple negative (*n *= 129)**		**Non-triple negative (*n *= 577)**		***P***^**a**^
	***N***	**%**	***N***	**%**	
Age at diagnosis, years					
≤50	61	47.29	247	42.81	0.354
>50	68	52.71	330	57.19	
Age at menarche, years					
≤13	22	17.05	95	16.46	0.494
14 to 16	57	44.19	256	44.37	
≥17	18	13.95	99	17.16	
Not available	32	24.81	127	22.01	
Childbearing history					
0 children	4	3.10	17	2.95	0.934
1 child	62	48.06	288	49.91	
2 children	28	21.71	117	20.28	
≥ 3 children	12	9.30	64	11.09	
Not available	23	17.83	91	15.77	
Tumor size					
≤2 cm	48	37.21	229	39.69	0.792
>2 cm	66	51.16	298	51.65	
Not available	15	11.63	50	8.67	
Lymph node involvement					
Negative	84	65.12	280	48.53	0.001
Positive	45	34.88	290	50.26	
Not available	0	0	7	1.21	
Family history					
Yes	8	6.20	30	5.20	0.599
No	110	85.27	512	88.73	
Not available	11	8.53	35	6.07	

^a^Chi-square test for age at diagnosis, tumor size, lymph node involvement, and family history distribution between triple negative and non-triple negative breast cancer patients; nonparametric rank test for distribution of age at menarche and childbearing between triple negative and non-triple negative breast cancer patients.

### Multivariate subgroup analysis of breast cancer risk in relation to family history

Details of the multivariate subgroup analyses of breast cancer risk in relation to family history are shown in Table 4. Compared with benign breast disease control, family history was not significantly associated with an increased risk of DCIS (OR = 1.27, 95% CI = 0.36 to 4.46, *P* = 0.704), while family history was significantly associated with an increased risk of invasive breast cancer (OR = 2.22, 95% CI = 1.32 to 3.75, *P* = 0.003) in our study.

**Table 4:** Risk of subtype-specific breast cancer in relation to family history of breast cancer compared with benign breast disease control

**Variable**	**OR (95% CI)**^**a**^	***P***^**a**^	**OR (95% CI)**^**b**^	***P***^**b**^
Pathology				
DCIS	1.27 (0.36 to 4.46)	0.704	1.30 (0.38 to 4.43)	0.679
Invasive cancer	2.22 (1.32 to 3.75)	0.003	2.26 (1.36 to 3.75)	0.002
Tumor size^c^				
≤2 cm	1.87 (0.92 to 3.80)	0.084	1.94 (0.97 to 3.87)	0.061
>2 cm	2.29 (1.22 to 4.29)	0.010	2.34 (1.28 to 4.30)	0.006
Lymph node involvement^c^				
Negative	2.60 (1.42 to 4.75)	0.002	2.56 (1.43 to 4.57)	0.001
Positive	2.07 (1.05 to 4.08)	0.036	2.14 (1.10 to 4.15)	0.025
Hormone receptor status^c^				
Negative	3.05 (1.45 to 6.41)	0.003	2.76 (1.35 to 5.64)	0.005
Positive	2.09 (1.16 to 3.78)	0.014	2.19 (1.23 to 3.89)	0.008
Molecular subtype^c^				
Triple negative	3.35 (1.43 to 7.88)	0.005	3.17 (1.38 to 7.28)	0.007
Non-triple negative	2.14 (1.21 to 3.80)	0.009	2.20 (1.27 to 3.82)	0.005

^a^Adjusted for age at diagnosis, age at menarche, and childbearing; ^b^Adjusted for age at menarche, and childbearing history; ^c^DCIS not included for analysis. CI, confidence interval; *DCIS*, ductal carcinoma *in situ*; OR, odds ratio.

In 803 invasive breast cancer patients, subgroup analyses regarding to tumor size, lymph node involvement, hormone receptor status, and molecular subtype were performed. When breast cancers were divided into two groups according to tumor size, family history of breast cancer was significantly associated with an increased risk of breast cancer >2 cm (OR = 2.29, 95% CI = 1.22 to 4.29, *P* = 0.010), and borderline significantly associated with an increased risk of breast cancer ≤2 cm (OR = 1.87, 95% CI = 0.92 to 3.80, *P* = 0.084). Furthermore, family history was significantly associated with an increased risk of breast cancer with negative (OR = 2.60, 95% CI = 1.42 to 4.75, *P* = 0.002) and positive lymph nodes (OR = 2.07, 95% CI = 1.05 to 4.08, *P* = 0.036). When breast cancers were divided into two groups according to hormone receptor status, family history was associated with an increased risk of both hormone receptor negative (OR = 3.05, 95% CI = 1.45 to 6.41, *P* = 0.003) and positive breast cancer (OR = 2.09, 95% CI = 1.16 to 3.78, *P* = 0.014). Importantly, having a family history of breast cancer was associated with an increased risk of triple negative breast cancer (OR = 3.35, 95% CI = 1.43 to 7.88, *P* = 0.005) with a magnitude of association similar to that for non-triple negative breast cancer (OR = 2.14, 95% CI = 1.21 to 3.80, *P* = 0.009). When multivariate analyses were performed without considering age at diagnosis, the results were similar to those given in Table 4.

## Discussion

This case–control study suggests that a family history of breast cancer is associated with an increased breast cancer risk; this is consistent with prior studies [7-12]. Importantly, the proportions of patients with a family history in the triple negative and non-triple negative groups were not significantly different in Han Chinese people. A family history of breast cancer was associated with an increased risk of triple negative breast cancer, with a magnitude of association similar to that for non-triple negative breast cancer in multivariate analyses. Furthermore, family history was not significantly associated with an increased risk of DCIS in our study.

Consistent with previous studies [3,7-12], our data indicate a 2.11-fold increased breast cancer risk in women with any-degree family history. However, subjects with benign breast disease, which is an important risk factor for subsequent breast cancer [8], were applied as controls. Furthermore, the proportion of subjects with a family history in the control group was 3.2%, while this proportion is about 1% in the general population in China [1]. So, the relationship between family history and risk of breast cancer may be underestimated in this case–control study.

In this study, triple negative tumors were observed among 18.3% of patients, a rate comparable to our previous study [26] and another Chinese study [28]. A lower proportion of lymph node involvement was observed in triple negative tumors in this study, which was also comparable to previous studies [26,28,29]. Even with less lymph node involvement, triple negative breast cancers still show poor prognosis. Thus, it is important to explore the risk of this subtype of tumors. In this study, we found a 3.55-fold increased risk of triple negative breast cancer in women with a family history, which was similar to that for non-triple negative breast cancer. Further cohort studies with large sample size are needed to confirm our findings.

Most previous studies are focused on the relationship between family history and risk of invasive breast cancers. However, DCIS cannot be ignored, although DCIS shows encouraging prognosis [30]. A long-term follow-up study [30] found that the rate of local recurrence was high, and 48% of these recurrences were invasive. Previous studies [21-25] suggest that family history is a risk factor of DCIS. However, no relationship between family history and risk of DCIS was observed in Han Chinese people. With the exception of this racial or ethnic difference, our results might also be influenced by small sample size and benign breast disease controls. The relationship between family history and risk of DCIS should be investigated in Asian populations to confirm our findings.

Several limitations still exist in our study. First, subjects with benign breast disease were selected as controls, so the association between family history and breast cancer risk may have been underestimated. Second, the sample size of DCIS in our study was relatively small; future studies with large sample sizes are needed to explore the relationship between family history and risk of DCIS. Third, owing to the nature of this case–control study, future large cohort studies should be conducted to explore these relationships.

## Conclusions

In conclusion, our results indicate that a family history of breast cancer is associated with an increased risk of triple negative breast cancer with a magnitude of association similar to that for non-triple negative breast cancer. Furthermore, family history was not significantly associated with an increased risk of DCIS in our study. Future cohort studies with large sample size are still needed to explore these relationships.

## Abbreviations

CI: Confidence interval; DCIS: Ductal carcinoma *in situ*; ER: Estrogen receptor; H & E: Hematoxylin and eosin; OR: Odds ratio; PR: Progesterone receptor; SD: Standard deviation.

## Competing interests

The authors declare that they have no competing interests.

## Authors’ contributions

SW contributed to the conception and design of the study, the analysis, and interpretation of data, the revision of the article, and final approval of the version to be submitted. KX, WZ, and HP participated in the design of the study, performed the statistical analysis, and drafted and revised the article. KX, WZ, HP, XiuL, ML, JinX, LC, JiaX, SC, XiaL, QD, and LL performed the study. All authors read and approved the final version of the manuscript.
